# Computational methods for ubiquitination site prediction using physicochemical properties of protein sequences

**DOI:** 10.1186/s12859-016-0959-z

**Published:** 2016-03-03

**Authors:** Binghuang Cai, Xia Jiang

**Affiliations:** Department of Biomedical Informatics, School of Medicine, University of Pittsburgh, Pittsburgh, PA 15206-3701 USA

**Keywords:** Ubiquitination, Ubiquitination Site Prediction, Protein sequence, Physicochemical property (PCP), Amino Acid (AA), Machine learning, Bayesian Network (BN), Support Vector Machine (SVM), Logistic Regression (LR), Least Absolute Shrinkage and Selection Operator (LASSO), Prediction

## Abstract

**Background:**

Ubiquitination is a very important process in protein post-translational modification, which has been widely investigated by biology scientists and researchers. Different experimental and computational methods have been developed to identify the ubiquitination sites in protein sequences. This paper aims at exploring computational machine learning methods for the prediction of ubiquitination sites using the physicochemical properties (PCPs) of amino acids in the protein sequences.

**Results:**

We first establish six different ubiquitination data sets, whose records contain both ubiquitination sites and non-ubiquitination sites in variant numbers of protein sequence segments. In particular, to establish such data sets, protein sequence segments are extracted from the original protein sequences used in four published papers on ubiquitination, while 531 PCP features of each extracted protein sequence segment are calculated based on PCP values from AAindex (Amino Acid index database) by averaging PCP values of all amino acids on each segment. Various computational machine-learning methods, including four Bayesian network methods (i.e., Naïve Bayes (NB), Feature Selection NB (FSNB), Model Averaged NB (MANB), and Efficient Bayesian Multivariate Classifier (EBMC)) and three regression methods (i.e., Support Vector Machine (SVM), Logistic Regression (LR), and Least Absolute Shrinkage and Selection Operator (LASSO)), are then applied to the six established segment-PCP data sets. Five-fold cross-validation and the Area Under Receiver Operating Characteristic Curve (AUROC) are employed to evaluate the ubiquitination prediction performance of each method. Results demonstrate that the PCP data of protein sequences contain information that could be mined by machine learning methods for ubiquitination site prediction. The comparative results show that EBMC, SVM and LR perform better than other methods, and EBMC is the only method that can get AUCs greater than or equal to 0.6 for the six established data sets. Results also show EBMC tends to perform better for larger data.

**Conclusions:**

Machine learning methods have been employed for the ubiquitination site prediction based on physicochemical properties of amino acids on protein sequences. Results demonstrate the effectiveness of using machine learning methodology to mine information from PCP data concerning protein sequences, as well as the superiority of EBMC, SVM and LR (especially EBMC) for the ubiquitination prediction compared to other methods.

**Electronic supplementary material:**

The online version of this article (doi:10.1186/s12859-016-0959-z) contains supplementary material, which is available to authorized users.

## Background

Ubiquitination (also known as ubiquitylation) is an enzymatic and post-translational modification process, in which ubiquitin (a small regulatory protein) is attached to a substrate protein [1–3]. In the process of ubiquitination, ubiquitin is bound to lysine (K) residues on the protein substrate via the three steps of activation, conjugation and ligation performed by ubiquitin activating enzymes (E1s), ubiquitin conjugating enzymes (E2s), and ubiquitin ligases (E3s), respectively [1–4]. Note that the binding can be either a single ubiquitin or chains of ubiquitin. Many regulatory functions of ubiquitination have been found, such as proteasomal degradation, DNA repair and transcription, signal transduction, and endocytosis and sorting, which are all important protein regulation functions in the biological processes [1–5].

Due to ubiquitination’s important regulation roles, research has been widely conducted to further decipher the mechanism of the ubiquitination process and its other regulatory roles at the molecular level. One of the initial and challenging steps towards gaining more understanding of ubiquitination is identification of ubiquitination sites. Different types of experimental methods have been employed to purify ubiquitination proteins in order to determine ubiquitination sites, such as high-throughput Mass Spectrometry (MS) techniques [6–9], ubiquitin antibodies and ubiquitin binding proteins [9, 10], and combinations of liquid chromatography and mass spectrometry [11]. However, the experiments that purify ubiquitination proteins are very time-consuming, expensive and labor-intensive, because the ubiquitination process is dynamic, rapid and reversible [4, 12, 13]. To reduce experiment cost and improve the effectiveness and efficiency of ubiquitination site identification, computational (*in silico*) methods have been introduced and developed based on informatics techniques for the prediction of ubiquitination sites based on prior knowledge of protein sequences [4, 5, 12, 13].

Machine learning methods have been applied to the protein ubiquitination site prediction problem. Various ubiquitination prediction methods, algorithms and tools have been developed that address different features of the protein sequences [4, 5, 11–16]. For example, Tung and Ho developed a tool called UbiPred, which uses the informative PhysicoChemical Property (PCP) mining algorithm to select informative PCP features, and then employs a support vector machine (SVM) for ubiquitination site prediction (Note that the SVM is selected based on the comparison of the performance of the SVM with naïve Bayes (NB) and k-nearest neighbor classifiers) [4]. Radivojac et al developed a random forest predictor for ubiquitination sites, named UbPred, which uses different sequence attributes including Amino Acid (AA) compositions and PCP [11]. A sequence-based predictor of ubiquitination site was developed based on a nearest neighbor algorithm using the features of PSSM (Position-Specific Scoring Matrix) conservation scores (quantifying the conservation status of each site in the protein sequence), AA factors, and disorder scores (disorder status of each site in the protein sequence) [13]. Chen et al developed an ubiquitination prediction tool called CKSAAP_UbSite based on the SVM technique and with composition of k-spaced AA pairs as the features [14]. They also developed a human-specific ubiquitination prediction tools, named hCKSAAP_UbSite, which integrates CKSAAP_UbSite with two other SVM classifiers (i.e., the one based on binary amino acid encoding and the one based on the AAindex [17] physicochemical property encoding) using Logistic Regression (LR) [15]. Chen et al presented a tool known as UbiProber for large scale predictions of ubiquitination based on SVM technique using three features of K nearest neighbor, AA composition, and PCP [16]. Walsh et al designed a sequence-based ubiquitination predictor called Rapid UBIquitination (RUBI) for rapid application on a genome scale using an iterative approach [5]. These studies and tools use different features/properties of the protein sequences as the data to learn and predict the ubiquitination sites, in which the protein sequences are obtained from different sources including research papers and experiments. Note that, among different types of features used for ubiquitination site prediction, PCP is one of the most important and widely used feature types, which influence the posttranslational modification process (including ubiquitination) and define the protein structures and functions together with biochemical properties [17, 18]. Thus, we will use the same information (i.e., PCPs) of protein sequences as the features for ubiquitination site prediction in this paper.

As mentioned above, different kinds of machine learning methods have been employed for the ubiquitination prediction model development, owing to their powerful model learning and prediction abilities. For example, Bayesian networks are a powerful probability-based method for model learning, which have been widely used in biomedical problems [19–24]. The SVM is a supervised learning model that finds the maximum margin hyper-plane separating the classes [25], and which has been used in many domains including biomedical informatics [4, 5, 14–16, 20, 26, 27]. Logistic Regression (LR) is another widely used regression analysis method based on the logistic function [28], which has also been popularly employed for prediction and classification in biomedical problems [15, 20, 29, 30]. Least Absolute Shrinkage and Selection Operator (LASSO) is a shrinkage linear method for regression widely used in prediction and classification, which is simple and can often describe the relation between the inputs and the output adequately and interpretably [31–33].

This paper aims at exploring different machine learning methods for ubiquitination site prediction using the same type of information of protein sequences, i.e., PCP features. Six segment-PCP data sets are first established based on the AAindex database [17, 18] and protein sequence data from the literature. Two categories (i.e., Bayesian-based and regression-based) machine learning methods are then applied to analyzing the six segment-PCP data sets for ubiquitination site prediction. Bayesian-based methods include Naïve Bayes (NB) [19, 20, 24, 34], Feature Selection NB (FSNB) [20, 34], Model Averaged NB (MANB) [34, 35], and Efficient Bayesian Multivariate Classifier (EBMC) [20, 36], while regression-based methods are SVM [25], LR [28], and LASSO [31–33]. We compared the performance of different prediction models for ubiquitination sites in terms of the values of the Area Under Receiver Operating Characteristic Curve (AUROC). Experimental results demonstrate the effectiveness of machine learning methods for mining information from protein sequence PCP data for ubiquitination prediction, as well as the superior performance of EBMC, SVM and LR (especially EBMC) as compared with other methods.

The remainder of this paper is organized in four sections. Section “Methods” presents the data establishing methods of the six segment-PCP data sets and the seven machine learning methods used for ubiquitination site prediction. In Section “Results”, experimental results are described and analyzed. A discussion appears in Section “Discussion”, and Section “Conclusions” concludes the paper with final remarks.

## Methods

Methods for establishing and processing the segment-PCP data are presented, and the machine learning methods (including NB, FSNB, MANB, EBMC, SVM, LR, and LASSO) for the ubiquitination site prediction based on the established segment-PCP data sets are then introduced.

### Data sets

**Fig. 1:**
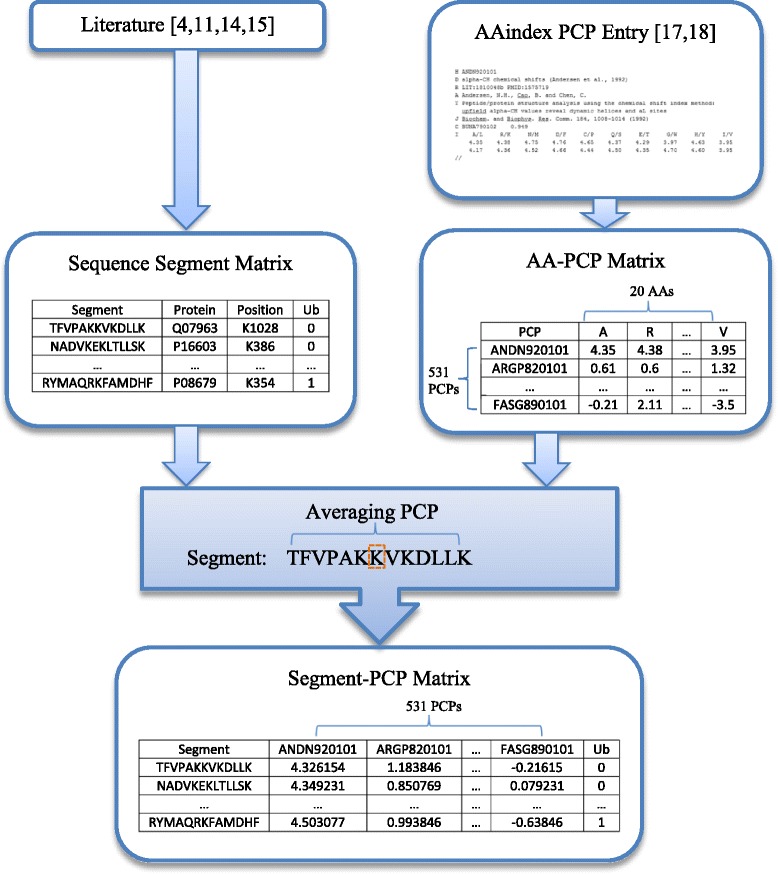
Diagram of the process for segment-PCP prediction matrix generation

As we know, a protein is a biological molecule that consists of one or more long chains of amino acid residues. A protein sequence is commonly built by 20 different amino acids (AAs) with 1-letter abbreviations as ARNDCQEGHILKMFPSTWYV [17, 18]. Among these AAs, lysine (K) is the essential amino acid that binds the ubiquitin and affects the protein function via ubiquitination [1–3]. To identify whether a lysine (K) is an ubiquitination site, we would need to gain information of the amino acids around the lysine (K) residue. To gather such information for the ubiquitination prediction, protein sequence segments are thus extracted from the long chain of AA residues for the ubiquitination prediction, while PCPs for each segment are calculated based on PCPs of each AA on the segment. The data establishing process includes the following three steps: sequence segment extraction, AA-PCP matrix generation, and segment-PCP prediction matrix generation. Details are shown in the diagram of Fig. 1, and are described next.

First, six data sets of protein sequences are collected from the literature, with the number of segments and data sources shown in Table 1. The detailed extraction process of each data set is described as below.

**Table 1:** Sequence segment data set list for ubiquitination site prediction

Data Set #	Number of Segments			Data Sources
	All	With	With Non-	
		Ubiquitination Central K Site		
1	300	150	150	[4] - http://iclab.life.nctu.edu.tw/ubipred/
2	6838	3419	3419	[15] (Independent Set) - http://protein.cau.edu.cn/cksaap_ubsite/download/DatasetForhCKSAAP_UbSite.rar
3	12236	6118	6118	[15] (Training Set) - http://protein.cau.edu.cn/cksaap_ubsite/download/DatasetForhCKSAAP_UbSite.rar
4	4608	263	4345	[14] - DOI: 10.1371/journal.pone.0022930.s001
5	3651	131	3520	[11] - http://www.ubpred.org/UbPred_DataSets.zip
6	676	37	639	[14] - DOI: 10.1371/journal.pone.0022930.s003

- In Data Set 1 (Set 1 in Additional file 1), a 300-sequence set is collected from [4], in which 150 sequence segments include ubiquitination lysine K sites in the center of the 13-AA segments (or termed, positive segments) and 150 segments have non-ubiquitination central K sites (or termed, negative segments).
- Data Set 2 (Set 2 in Additional file 1) with 6838 27-AA segments is from the independent data set in [15], which has half-half ubiquitination and non-ubiquitination K sites in the center of the protein sequence segments.
- In Data Set 3 (Set 3 in Additional file 1), 12236 27-AA protein sequence segments are obtained from the training data set in [15], with 50 % of the segments having ubiquitination central K sites and 50 % having non-ubiquitination central K sites.
- In Data Set 4 (Set 4 in Additional file 1), we have 4608 27-AA protein sequence segments (containing 263 ubiquitination ones and 4345 non-ubiquitination ones), which are extracted from all the K sites in 203 proteins (with sequences stored in FASTA format) containing experimentally validated ubiquitination sites used in [14].
- In Data Set 5 (Set 5 in Additional file 1), we extracted all K sites from 96 protein sequences (in FASTA format) collected from experiments and literature by the authors in [11], which results in 3651 27-AA segments with 131 ubiquitination positive segments and 3520 non-ubiquitination negative ones.
- In Data Set 6 (Set 6 in Additional file 1) is a set of 676 27-AA protein sequence segments (with 37 ubiquitination ones and 639 non-ubiquitination ones, and all with central K sites), which is extracted from 21 protein sequences (in FASTA format) containing reportedly verified ubiquitination sites that were used as an independent testing data in [14].

Each row of the six protein sequence segment matrices (with illustrative example shown in the second block on the left of Fig. 1) contains the segment sequence, the protein (entry name/ID), the position of the central K in the original protein sequence, and the ubiquitination class (1 as having ubiquitination central K site, and 0 as non-ubiquitination central K site). Note that, in the process of segment extraction, if the K site is near the beginning or the end of a protein sequence, appropriate number of “-“ symbols are appended to the beginning or the end of the segment in order to form each segment with the same length. In addition, every protein sequence segment in the six data sets contains K sites in the center of the segment, which could be either ubiquitination or non-ubiquitination sites. The six extracted protein sequence segment matrix data sets are included in the Additional file 1.

Second, due to the importance of PCP of amino acid in protein sequence function, PCPs of each AA on the sequence segment are collected from AAindex [17, 18] for the ubiquitination site prediction. The PCP data in AAindex1 format is processed to create an AA-PCP matrix with each column being the PCP value of each of the 20 AAs and each row being a PCP feature. In such an AA-PCP data set, a row title is the AAindex entry accession number of the PCP, and a column title is 1-letter abbreviation of the 20 AAs. Since 13 PCP features with missing values are deleted, there are 531 PCP features (rather than 544 PCPs included in AAindex) in the AA-PCP matrix used in this paper. An example of the AA-PCP matrix is shown in the second block on the right in Fig. 1. Note that biological descriptions and related studies of each PCP can be found though AAindex database [17, 18].

Finally, based on the six protein sequence segment data sets and the AA-PCP matrix, we established six segment-PCP matrices for the ubiquitination site prediction. In such matrices, each row is a protein sequence segment and each column is a PCP feature, as shown in the bottom block of Fig. 1. The PCP values are calculated based on averaging. That is, to get the value of a particular PCP feature for a particular segment, all AA values for this PCP for all the AAs on the segment are averaged. There are totally 531 PCP features/columns in each segment-PCP data set, while the numbers of the segments/rows of each of the six data sets are the same as the ones shown in second column of Table 1. The ubiquitination class is added as the last column in the data sets. Sequence segments and PCP accession numbers are shown as the row title and column title, respectively. Therefore, we get six different segment-PCP data sets for the ubiquitination site prediction.

In summary, six ubiquitination prediction data sets are established based on the protein sequences from different literature sources and the PCP values from AAindex, which include three balanced data sets (i.e., data sets with same numbers of ubiquitination and non-ubiquitination samples) and three unbalanced data sets (i.e., data sets with different numbers of ubiquitination and non-ubiquitination samples). These data sets are used to evaluate the performance of different machine learning methods for the ubiquitination site prediction.

### Bayesian-based methods

A Bayesian network is a probabilistic graphical model consisting of a Directed Acyclic Graph (DAG) *G* = (*V*, *E*), which has been widely used for machine learning and uncertain reasoning in many areas, including bioinformatics [19, 20, 37–39]. In a Bayesian network, the nodes *V* represent random variables and the edges *E* represent the probabilistic relationships (i.e., conditional independencies) among the nodes. A Bayesian network has a conditional probability distribution of each node given each combination of values of its parents, which represents conditional independencies among nodes. Four different types of Bayesian network methods are employed for the ubiquitination site prediction based on the six segment-PCP data sets established in the above subsection. These Bayesian methods include NB [19, 20, 24, 34], FSNB [20, 34], MANB [34, 35], and EBMC [20, 36]. Details of the applications of such Bayesian methods to ubiquitination prediction are described as follows.

**Fig. 2:**
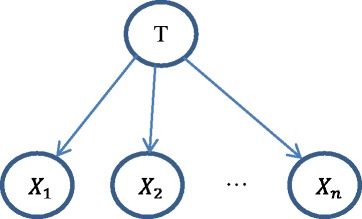
DAG model of naïve Bayes

NB is an ideal, simple, and widely-used Bayesian network model with all the features/variables {*X*_*i*_, *i* = 1, 2, ⋯, *n*} as children of the target, which shows all the features of interest to the target outcome [19, 20, 24, 34]. The NB model assumes that the variables (PCP features in this paper) for prediction are independent of each other conditioning on the value of the target. An illustrative DAG for a NB network is shown in Fig. 2. To use the NB network for the ubiquitination site prediction, we calculate the following probability function.

$$ P\left(T\Big|{X}_1,{X}_2,\cdots, {X}_n\right)=\gamma P\left({X}_1,{X}_2,\cdots, {X}_n\Big|T\right)P(T)=\gamma {\displaystyle {\prod}_{i=1}^nP\left({X}_i\Big|T\right)P(T)}, $$

where *γ* is a scaling factor (or termed, normalizing constant). Given the 531 PCP features of a segment, the NB classifier provides the probability of the state of the target (i.e., whether the central K site is ubiquitination site or not).

FSNB [20, 34] is a Bayesian prediction method based on feature selection and NB. The method starts with no feature in the model and then uses a greedy search to add the feature to the model that most increases the Bayesian score introduced by Cooper and Herskovits in [40]. If no additional feature increases the score, the search stops. The final model will be used for the prediction where the features included in the model are the selected predictors. FSNB can greatly reduce the computational complexity of the Bayesian network, especially for large-scale data with many features/variables.

MANB is a Bayesian network prediction method based on model averaging and NB [34, 35]. MANB calculates the probability of the state of the target based on the NB model containing each subset of all the PCP features and then averages the probabilities over all subsets. Since it is unfeasible to calculate the probabilities for all the 2^*n*^ subsets for large numbers of features, algorithms have been developed by exploiting the conditional independencies in [34, 35, 41], which reduce the computational complexity from *O*(2^*n*^) to *O*(*n*). MANB can therefore handle all the subsets of the 531 PCP features.

EBMC is an efficient Bayesian classifier recently-developed by Cooper et al [36], which is based on scoring and search techniques similar to FSNB but in a refined manner [20, 36]. EBMC is summarized as the following steps.

1. EBMC starts from an empty Bayesian network model and searches for the variable (i.e., a PCP feature) that best predicts the target (i.e., ubiquitination or non-ubiquitination central K site) based on the supervised scoring method [42] in conjunction with the BDeu scoring measure [43].
2. EBMC then searches for a second variable that, when combined with the first variable found, best predicts the target.
3. This procedure is done iteratively until no additional variable, when combined with the existing variables, better predicts the target. The model obtained is then converted to a statistically equivalent model in which the variables are children of the target.
4. EBMC then proceeds to add variables as parents of the target in the generated model, repeating steps (2) and (3).
5. EBMC proceeds in this manner until prediction cannot be improved.

**Fig. 3:**
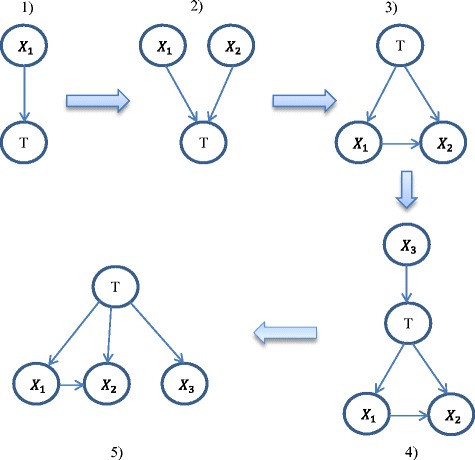
Illustrative example of EBMC

An illustrative example of each step of the above procedure of EBMC is shown in Fig. 3. In the figure, Node “T” is the target (i.e., ubiquitination site class) and Nodes “ *X*_1_”, “ *X*_2_” and “*X*_3_” are the variables (i.e., PCP features). A detailed procedure for EBMC is described in [20, 36]. EBMC has been employed for biomedical prediction based on different data sets including simulated SNP data and real genome data, and has been shown to be effective and efficient for the prediction problem [20, 36].

### Regression-based methods

Besides the four Bayesian methods mentioned above, we also used three other machine learning methods for the ubiquitination site prediction based on the segment-PCP data. The methods include SVM, LR and LASSO, which are all regression-based machine learning methods.

SVM is a non-probabilistic machine learning method, which tries to separate the classes by finding the maximum margin hyper-plane [25]. In particular, for the ubiquitination site prediction problem, given the training segment-PCP data {(***x***_*i*_, ***y***_*i*_)|***x***_*i*_ ∈ *R*^531^, ***y***_*i*_ ∈ {0, 1}, *i* = 1, 2, ⋯, *N*} with ***y***_*i*_ indicating the ubiquitination class to which the point ***x***_*i*_ belongs and each ***x***_*i*_ being a 531-dimensional real vector of the PCP values of the *i*th protein sequence segment, SVM finds the maximum-margin hyper-plane that divides the points with ***y***_*i*_ = 1 from those with ***y***_*i*_ = 0. Any hyper-plane can be written as the set of points ***x*** satisfying ***w***^*T*^***x ***- *b* = 0 with ***w*** being the weighting parameter vector and *b* is a constant parameter. When we solve the equation and get the weight ***w***, we then have the model for prediction.

LR is another type of regression analysis method used for predicting the outcome of a target based on one or more feature variables [28]. In this paper, LR describes the probability of the possible state outcome of the target (i.e., central K site) by modelling it as a logit function:

$$ logit\left(\ P\left({\boldsymbol{y}}_i\Big|{\boldsymbol{x}}_i\right)\right)={\beta}_0{x}_{0i}+{\beta}_1{x}_{1i}+\cdots +{\beta}_k{x}_{ki}+\cdots +{\beta}_{531}{x}_{531i}, $$

where $logit\left(\pi \right)= \log \left(\frac{\pi }{1-\pi}\right),\;\left\{\left({\boldsymbol{x}}_i,{\boldsymbol{y}}_i\right)\Big|i=1,2,\cdots, N\right\}$ is the *i*th segment of segment-PCP data and target label (ubiquitination or non-ubiquitination), *β*_*k*_ is the *k*th parameter of the model, and *x*_*ki*_ is the value of the *k*th PCP feature in the *i*th segment (with *x*_0*i*_ = 1). We can use maximum likelihood estimation to get the parameters so as to maximize the probability of the segment-PCP data [28]. When the parameter *β*_*k*_ is established, we can use the model to estimate the probability of the prediction target via

$$ P\left({\boldsymbol{y}}_i\Big|{\boldsymbol{x}}_i\right)={e}^t/\left(1+{e}^t\right)\kern0.5em \mathrm{with}\ t={\beta}_0{x}_{0i}+{\beta}_1{x}_{1i}+\cdots +{\beta}_k{x}_{ki}+\cdots +{\beta}_{531}{x}_{531i.} $$

LASSO is a shrinkage linear regression method. For ubiquitination site prediction in this paper, LASSO fits the linear model for the target of the *i*th segment in the segment-PCP data as

$$ {\widehat{y}}_i={b}_0+{b}_1{x}_{1i}+{b}_2{x}_{2i}+\cdots +{b}_k{x}_{ki}\cdots +{b}_{531}{x}_{531i}, $$

where *x*_*ki*_ is the *k*th PCP value of the *i*th segment, *b*_0_ is a constant, and *b*_1_, ⋯, *b*_*k*_, ⋯, *b*_531_ are the model parameters for each of the 531 PCP features. The solution is obtained by min ∑_*i* = 1_^*N*^(*y*_*i*_ − *ŷ*_*l*_)^2^, s. t. ∑_*k* = 0_^531^|*b*_*k*_| ≤ *s* with *s* being the bound tuning parameter [31–33]. We see that solving the LASSO is a Quadratic Programming (QP) problem. When the QP problem is solved and parameters *b*_0_, *b*_1_, ⋯, *b*_531_ are obtained, we can use the model for ubiquitination site prediction.

### Experimental method

In the experiments, Java (http://www.java.com/en/) is employed as the programing language to create the six segment-PCP matrix data sets from the different formats of the sequence data from different sources based on the descriptions in the “Data sets” subsection. Discretization is also applied on the segment-PCP data sets in Java so that Bayesian-based machine learning methods can be used on the data. In each segment-PCP dataset, the discretization is based on the values of each PCP feature (i.e., each column in the segment-PCP data sets), which are divided into three equal-interval ranges. Each PCP value is replaced by one of the three discreet values (i.e., 0, 1, 2) based on the range in which it belongs.

For the seven machine learning methods, Java and MATLAB [44] are both used to implement the methods for ubiquitination site prediction. In particular, the Bayesian-based methods (i.e., NB, FSNB, MANB, and EBMC, see [20, 34–36, 40]) are implemented in Java, and the EBMC Java package we used is now an official package in Weka (https://weka.wikispaces.com); SVM and LR are implemented in MATLAB using the LIBLINEAR [45] package; and LASSO is implemented in Java using the “lasso4j” (http://code.google.com/p/lasso4j/) package. We conducted experiments in the eclipse environment (http://www.eclipse.org/) for the Java implementations, and in the MATLAB 2013a environment [44] for the MATLAB implementations. Experiments were conducted on a Dell PowerEdge R515 server which has a 2.80 GHz (2 processors) AMD Opteron 4280 and 128G RAM memory. Note that in the experiments concerning SVM and LR using LIBLINEAR, we used different penalty parameters by setting parameter “-wi” for different classes (i.e., ubiquitination or non-ubiquitination) in order to handle large differences in the numbers of cases in the two classes (i.e., unbalanced Data Sets 4-6 in Additional file 1) [45].

To evaluate the performance of different methods for ubiquitination site prediction, the AUROC is used as the performance criterion, while *p*-value and outperformance percentage are also used to compare the AUROCs between methods. Note that the *p*-value is obtained based on *t*-test by using MATLAB function “ttest” [44]. Five-fold cross-validation [46] is employed in the experiment to get a testing AUROC for each segment-PCP data set. Specifically, each segment-PCP data set is divided into five parts based on the proportion between ubiquitination and non-ubiquitination in the whole segment-PCP data set. In each fold, one part of the five parts of the data set was selected as the testing data while the remaining four parts were considered as the training data [33]. This is repeated for all five parts and the final AUROC is the average value of all AUCs of five folds. In addition, computational time is also presented to evaluate the efficiency of the presented ubiquitination site prediction methods, which is calculated as the total running time used to conduct all the five folds of cross-validation, including all training and testing times for the prediction.

## Results

**Fig. 4:**
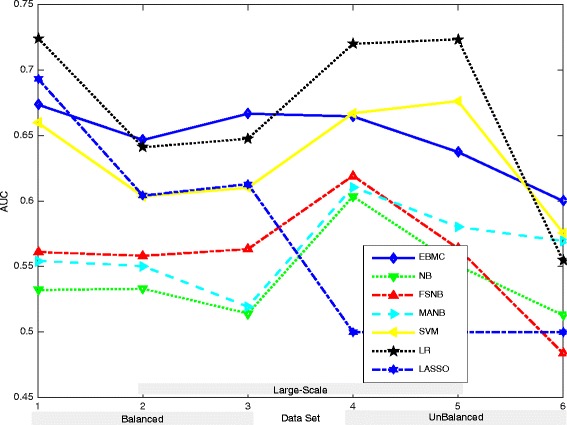
AUROC comparison of different machine learning methods for ubiquitination prediction using different data sets

In this section, the experiment results for different methods for ubiquitination site prediction are presented, described, and compared based on segment-PCP data. The results are shown in Tables 2, 3 and 4 and Fig. 4.

**Table 2:** AUROC of ubiquitination prediction results from different methods based on different segment-PCP data sets

Data Set #	EBMC	NB	FSNB	MANB	SVM	LR	LASSO
1	0.6714	0.5289	0.5613	0.5545	0.6597	0.7244	0.6933
2	0.6467	0.5330	0.5582	0.5502	0.6035	0.6410	0.6041
3	0.6667	0.5141	0.5633	0.5192	0.6102	0.6476	0.6129
4	0.6646	0.6036	0.6193	0.6108	0.6670	0.7200	0.5000
5	0.6373	0.5505	0.5637	0.5804	0.6763	0.7235	0.5000
6	0.6001	0.5134	0.4838	0.5690	0.5758	0.5546	0.5000

**Table 3:** Statistical analysis and comparisons between EBMC and other methods for ubiquitination site prediction

Data Type		All	Balanced	UnBalanced	Large-scale
EBMC : NB	Outperformance %	20.12	25.87	14.25	19.22
	*p*-Value	0.0007	0.0072	0.0118	0.0132
EBMC : FSNB	Outperformance %	16.37	18.09	14.80	13.65
	*p*-Value	0.0004	0.0047	0.0628	0.0082
EBMC : MANB	Outperformance %	15.18	22.47	8.03	16.14
	*p*-Value	0.0056	0.0146	0.0284	0.0271
EBMC : SVM	Outperformance %	2.77	6.17	−0.64	2.57
	*p*-Value	0.3108	0.0950	0.7854	0.5527
EBMC : LR	Outperformance %	−2.48	−1.06	−3.80	−3.94
	*p*-Value	0.3687	0.7238	0.5051	0.3268
EBMC : LASSO	Outperformance %	15.51	4.32	26.80	19.05
	*p*-Value	0.0359	0.3800	0.0189	0.0461

**Table 4:** Computational time (seconds) of ubiquitination predictions by different methods for different segment-PCP data sets

Data Set #	EBMC	NB	FSNB	MANB	SVM	LR	LASSO
1	5.039	1.232	2.794	4.385	0.785	0.412	1.147
2	270.115	9.220	571.632	43.486	34.763	17.946	3.384
3	586.531	11.045	1936.617	46.816	42.151	18.622	5.152
4	78.142	6.443	138.030	30.780	5.249	5.746	3.337
5	52.869	3.775	44.711	19.314	39.540	15.836	2.011
6	6.928	1.700	4.166	6.662	10.229	5.273	1.126

Table 2 and Fig. 4 show the testing AUROC of the ubiquitination site prediction outcome of each method for each segment-PCP data set. From the table and the figure, we can see that most of the methods obtain AUROCs between 0.51 to 0.73 for the six segment-PCP data sets. This shows that the segment-PCP data contain useful information that could be used for the ubiquitination site prediction, and also demonstrates the effectiveness of different machine learning methods for mining information from the segment-PCP data for the prediction.

From Table 2 and Fig. 4, we can find that EBMC, SVM and LR perform similarly with each other in one tier for most of the six segment-PCP data sets, and the three other Bayesian methods (i.e., NB, FSNB and MANB) perform similarly with each other in another tier. However, the former group has better AUROCs than the latter one. Also, we can see that EBMC and LR have better AUROC values than SVM for most of the data sets (except EBMC for Data Set 5 in Additional file 1 and LR for Data Set 6 in Additional file 1, with slightly smaller AUROCs than SVM). For LASSO, we can see that it has similar AUROC values with the first group for balanced data sets (i.e., Data Sets 1-3 in Additional file 1), while it has all 0.5 AUCs for the three unbalanced data sets (i.e., Data Sets 4-6 in Additional file 1). This shows that LASSO could perform well with balanced data but not with unbalanced data for the ubiquitination site prediction. In a word, EBMC, LR and SVM have better performance for prediction than other methods. In addition, EBMC, LR and SVM could also perform better than some of the existing ubiquitination prediction tools like UbiPred [4], CKSAAP_UbSite [14] and UbPred [11] (with AUCs being 0.6466 (EBMC), 0.6410 (LR), 0.6035 (SVM) as compared to 0.560 (UbiPred), 0.467 (CKSAAP_UbSite), 0.497 (UbPred)), as shown in Fig. 3 of [15] for the same protein sequence set (i.e., the independent set of [15]) as Data Set 2 in Additional file 1 of this paper. Also, EBMC even performs better than UbPred (with AUC as 0.6001 compared to 0.5872) for the same protein sequence set (i.e., the independent set of [14]) as Data Set 6 in Additional file 1 of this paper, as shown in Fig. 4 of [14].

From Table 2 and Fig. 4, we can also see that EBMC is the only method that has all the AUROCs greater than or equal to 0.60 for all the six data sets, which shows its superiority for ubiquitination site prediction, as compared with the other six machine learning methods. Moreover, from the table and the figure together with Table 1, we see that EBMC can get increasing AUROCs as the size (i.e., the number of segments) of the data set increases, especially for unbalanced Data Sets 4-6 in Additional file 1. In particular, the AUROC for EBMC prediction outcome increases from 0.6000 to 0.6646 as the size of data set increases from 676 segments (Data Set 6 in Additional file 1) to 4608 segments (Data Set 4 in Additional file 1). We do not observe something like this in the AUROC results for SVM and LR. Also, we see that EBMC gets the best AUROC of 0.6667 for the biggest data set (i.e., Data Set 3 in Additional file 1 with 12236 segments), among all the seven machine learning methods. This result illustrates the prediction capacity of EBMC for large-scale data. That is, EBMC tends to perform better for larger data sets. In addition, we have also checked the sensitivity values at 10 % false positive rate control for EBMC predicted outcomes. Such values for EBMC are between 0.1889 and 0.3502, which are significantly better than the random guessing rate (0.1). This shows the effectiveness of EBMC for ubiquitination prediction even at low false positive rate control.

Table 3 shows the statistical analysis (including outperformance percentage and *p*-value) of the AUROC results between EBMC and the other six machine learning methods for four groups of segment-PCP data sets (i.e., “All” -- all six data sets, “Balanced” -- the three balanced data sets, “UnBalanced” -- the three unbalanced data sets, and “Large-Scale” -- the four large-scale data sets with more than 3500 segments, i.e., Data Sets 2-5 in Additional file 1). On the one hand, we can see that EBMC significantly outperforms NB, FSNB, MANB and LASSO by more than 15 % with *p*-value smaller than 0.05 based on the AUROC results for all six data sets. For example, EBMC outperforms NB by 20.12 % with *p*-value being 0.0007, and EBMC outperforms LASSO by 15.51 % with *p*-value being 0.0359. For the three balanced data sets, EBMC also significantly outperforms other three Bayesian methods by more than 18 %. For instance, EBMC outperforms NB by 25.87 % with *p*-value being 0.0072. For the three unbalanced data sets, EBMC also significantly performs by more than 8 % relative to NB, MANB and LASSO (e.g., 26.80 % better than LASSO with *p*-value = 0.0189). For the four large-scale data sets, EBMC performs significantly better than the other three Bayesian-based methods and LASSO as well, with outperformance percentages more than 13 % and *p*-values less than 0.05.

On the other hand, from Table 3, we see that the outcomes for EBMC are comparable with SVM and LR. The outcome for EBMC for balanced data sets is slightly (6.17 %) better than SVM, while the outcome for SVM for unbalanced data sets is slightly (0.64 %) better than EBMC. The overall outcome of EBMC is slightly (2.77 %) better than SVM for the four different groups of data sets. We also see that LR performs slightly (less than 4 %) better than EBMC. However, all the *p*-values of these comparisons are much bigger than 0.05. Based on these results, it is hard to say which of these three methods (i.e., EBMC, SVM and LR) significantly performs better than others, but we could say that they are comparable for ubiquitination site prediction.

In addition, Table 4 shows the computational time (i.e., the total running time used to conduct the five-fold cross-validation, including all training and testing times) of the seven different machine-learning methods for ubiquitination predictions for the six data sets. The table demonstrates that the computational time is reasonable for practical use of ubiquitination site prediction, since the maximal computational time is less than 33 min (i.e., 1936.617 s for the 12236-segment Data Set 3 in Additional file 1 using FSNB). From the table, we can see NB and LASSO use a few seconds to finish the calculation of prediction, while MANB, SVM and LR take less than a minute to do the training and prediction for any of the six data sets. EBMC and FSNB seem to take more time to finish the computations in many cases. However, EBMC could be more efficient than other methods in some cases. For example, for the 676-segment small data set (i.e., Data Set 6 in Additional file 1), EBMC takes 6.928 s to finish the calculation, which is less than the 10.229 s required by SVM. For the largest data set (i.e., Data Set 3 in Additional file 1 with 12236 segments), EBMC takes 586.531 s (less than 10 min) for the computation, which is about ¼ of the time (i.e., 1936.617 s) used by FSNB. All these demonstrate the efficiency of the seven methods (including EBMC) for ubiquitination site prediction using segment-PCP data, including for large-scale data.

In summary, the experimental results show the effectiveness and efficiency of the presented seven machine learning methods for mining information from PCP data of protein sequences in order to predict ubiquitination sites. Results also demonstrate that EBMC, SVM and LR perform better than other methods, while EBMC tends to perform better for large-scale data (especially for unbalanced ones) as compared with other methods. These results could be helpful for the development of new ubiquitination prediction tools.

## Discussion

According to the experiment results for six different segment-PCP data sets described in the previous section, we see that PCP is an important biological property of protein sequences, which can be used for ubiquitination site prediction. Also, the PCP averaging strategy over all the AAs on the segment around central lysine K site is an effective way to gain information from each protein site for ubiquitination prediction. Although the PCP averaging strategy has been shown to be effective for ubiquitination site prediction, whether there are better strategies to summarize PCP values over the sequence that could improve the prediction is a very interested topic for future study. Moreover, since we used all the 531 PCPs as features for the ubiquitination prediction in this paper, whether there is a subset of the 531 PCPs that could be more informative for the prediction is another research topic that could be further investigated.

In this paper, the lengths of the protein sequence segments are fixed (i.e., 27 for most of data sets, and 13 for Data Set 1 in Additional file 1 due to the availability of the source protein sequences). Can different lengths of protein segments affect the prediction outcome? Is there an optimal segment length for ubiquitination site prediction? Do longer segments contain more information for prediction? These are all interesting questions that can be further investigated. Optimal length selection methods might be developed for protein site prediction to address these questions.

The experimental results demonstrated the effectiveness of the seven presented machine learning methods for ubiquitination site prediction using PCP data. When initially conducting the experiments for SVM, LR, and LASSO, we found that these three methods could not handle unbalanced data sets (i.e., Data Sets 4-6 in Additional file 1) as evidenced by their 0.5 AUC outcomes. This is because they over-fit the major class (i.e., the one with more segments, namely the non-ubiquitination class in this paper). All four Bayesian network methods don’t have this over-fitting problem, which shows the superiority of Bayesian network methods to handle unbalanced data, as compared with other regression methods. To deal with the over-fitting problems of SVM and LR, we set different penalty values for the major class and the minor class as mentioned in Subsection “Experimental method”. By doing this, SVM and LR get comparable results with EBMC, which are better than other presented methods. Note that the overall performance of EBMC is still slightly better than that of SVM, and EBMC also tends to perform better than SVM and LR for large-scale data sets. However, these comparisons are not significant. Also, the experiment results show that EMBC may take longer computational time for the ubiquitination prediction as compared with other methods. Thus, EBMC can be further improved to try to get significantly better prediction outcome and reduce computational time when comparing with traditional machine learning methods, such as SVM and LR.

In addition, the paper focuses on the ubiquitination site (i.e., lysine K site) prediction. The other 19 AAs are also important components of proteins and have their own biological functions. The presented data processing approaches and prediction methods can also be extended for the prediction of other protein AA sites. Extension of ubiquitination site prediction methods to strategies for other protein site prediction is also an interesting future research topic.

## Conclusions

We established six segment-PCP data sets for ubiquitination site prediction based on PCP information from AAindex and protein sequences from different literature sources via an averaging technique. To mine information from the six established data sets for prediction, seven machine learning methods including Bayesian-based and regression-based methods (i.e., NB, FSNB, MANB, EBMC, SVM, LR and LASSO) have been presented, compared and evaluated by cross-validation and AUROC criterion. The computationally experimental results show that PCP over the protein sequence segment is a useful type of feature for ubiquitination site prediction. The results also demonstrated the effectiveness of the presented machine learning methods for mining information from segment-PCP data for prediction. Comparisons illustrated the superior performance of EBMC, SVM and LR (especially EBMC for large-scale data) for ubiquitination site prediction, as compared with other methods. Comparison results could be useful for development for a new ubiquitination prediction tool. Future research may lie in the strategy of summarizing PCP over protein sequence segments, the informative PCP feature selection algorithm, the optimal length selection method of protein sequence segments, improvement of the EBMC algorithm, extension of the presented data processing and prediction approaches to the prediction of other protein sites, and development of future protein site prediction tools. (Note that links to software packages and dataset we used in this study are included in Additional file 2).

## Additional files

Additional file 1: Protein sequence segment data files. (ZIP 529 kb) Additional file 2: Web-links to databases and software packages used in this paper. (DOCX 14 kb)
