# Supplementary material for: Computational methods for ubiquitination site prediction using physicochemical properties of protein sequences
Source: BMC Bioinformatics. 2016 Mar 3;17:116. doi: 10.1186/s12859-016-0959-z (PMC4778322; doi:10.1186/s12859-016-0959-z)
Supplement: Additional file 2: — Web-links to databases and software packages used in this paper. (DOCX 14 kb) [file 12859_2016_959_MOESM2_ESM.docx]

**Supplement 2**

**AAindex (Amino Acid index database)**

( http://www.genome.jp/aaindex/)

**Methods and Software Packages Used in this Study**

**Bayesian network methods**

Naive Bayes (NB) (via Java package from [Dr. Gregory Cooper](http://www.dbmi.pitt.edu/person/gregory-cooper-md-phd) group)

Feature Selection NB (FSNB) (via Java package from [Dr. Gregory Cooper](http://www.dbmi.pitt.edu/person/gregory-cooper-md-phd) group)

Model Averaged NB (MANB) (via Java package from [Dr. Gregory Cooper](http://www.dbmi.pitt.edu/person/gregory-cooper-md-phd) group)

Efficient Bayesian Multivariate Classifier (EBMC)) (via Java package from [Dr. Gregory Cooper](http://www.dbmi.pitt.edu/person/gregory-cooper-md-phd) group; Java official package available in [Weka](https://weka.wikispaces.com))

**Regression methods**

Support Vector Machine (SVM) (via [LIBLINEAR](https://www.csie.ntu.edu.tw/%7Ecjlin/liblinear/) package in MATLAB)

Logistic Regression (LR) (via [LIBLINEAR](https://www.csie.ntu.edu.tw/%7Ecjlin/liblinear/) package in MATLAB)

Least Absolute Shrinkage and Selection Operator (LASSO) (via [lasso4j](http://code.google.com/p/lasso4j/) package in Java)
